# *La fantasma de Valencia* y las comedias de *Fiestas del jardín* de Alonso de Castillo Solórzano: hipótesis de datación y circunstancias de representación

**DOI:** 10.1007/s11061-021-09718-1

**Published:** 2022-01-17

**Authors:** Clara Monzó Ribes, Félix Blanco Campos

**Affiliations:** 1 Universidad de Viena, Vienna, Austria; 2 Universidad de Valladolid, Valladolid, Spain

**Keywords:** Alonso de Castillo Solórzano, *La fantasma de Valencia*, Juan Jerónimo Valenciano, *Fiestas del jardín*, Early Modern Spanish Theater

## Abstract

Alonso de Castillo Solórzano, prolific cultivator of the novelistic genre, gathered and published his work in miscellaneous volumes or *colectáneas* all along the XVII century. In these volumes, mainly formed by novels assembled in a narrative framework, dramatic pieces appear from time to time. This is the case of *Fiestas del Jardín* (1634), which alternates four plays written in prose and three comedies. We know about the last ones that they were brought to the stage by renowned authors of the time. However, the date of their composition remains unknown as well as the circumstances in which they were staged. It is our goal to shed light on these questions following the study of *La fantasma de Valencia*, one of the three aforementioned comedies.

Ciertas comedias de Castillo Solórzano se habían a buen seguro llevado a las tablas cuando, en 1634, aparecieron insertas en un volumen misceláneo: *Fiestas del jardín.* Tal noticia, nada inusual en el contexto del teatro áureo, sirve como punto de partida, en este caso, para conocer la fortuna que vivieron los dramas de don Alonso. Como señala Arredondo (2006, 36): ‘las “comedias representadas” constituyen uno de los interrogantes en la carrera de Castillo, porque conocemos los textos de las comedias intercaladas en sus obras narrativas, pero apenas nada de sus representaciones’. Entre el conjunto de comedias que se publicaron junto a los escritos en prosa —género que, a lo largo de su ubérrima producción, cultivó con indiscutible comodidad y preferencia—, solo tres nos llegan con visos de haber pisado la escena: *Los encantos de Bretaña*, *La fantasma de Valencia* y *El marqués del Cigarral*. En el prólogo a *Fiestas del jardín*, el propio dramaturgo nos informa ufano de que ‘ya han granjeado aplausos en los teatros de España’.^1^ Así, el lector que sostuviera entre sus manos el volumen tendría ocasión de disfrutar un vario entretenimiento y saltar a su gusto entre cuatro novelas —*La vuelta del ruiseñor*, *La injusta ley derogada*, *Los hermanos parecidos* y *La crianza bien lograda—* y tres comedias, hábilmente engarzadas en un armazón narrativo.^2^

No era la primera ocasión en que Castillo empleaba esta fórmula editorial. *Huerta de Valencia* (1629) o *Tiempo de regocijo* (1627) reproducen el mismo formato misceláneo, aunque en ellos ‘el peso de lo dramático se antoja poco significativo’ (Fuentes Nieto 2019, 10).^3^ No obstante, la ausencia de noticias, sumada a las características genéricas de las obras —a menudo supeditadas al entramado narrativo del marco en que se insertan—, apunta a que se trata de textos concebidos para la lectura antes que para las tablas (Festini, 2011, 223).^4^ La complejidad escenotécnica de alguna de estas comedias, con *La torre de Florisbella* a la cabeza, generosa en tramoya, las vuelve difícilmente representables (Soons, 1978, 39). Junto a estas piezas, están aquellas que, por motivos azarosos, no alcanzaron las tablas. Es el caso de *El mayorazgo figura*, obra que, a pesar de contar con licencia de representación fechada en 1638, termina por publicarse dentro de *Los alivios de Casandra* en 1640 sin alusión alguna a una puesta en escena previa.^5^

En cualquier caso, si hemos de creer a Castillo, lo cierto es que al menos las tres comedias de *Fiestas del jardín* atravesaron los tablados españoles. Tomando como punto de partida las palabras del dramaturgo y siguiendo el eco de aquellos aplausos, en estas páginas propondremos una hipótesis de datación de uno de los textos dramáticos reunidos en el volumen: *La fantasma de Valencia*.^6^ La pesquisa documental permitirá arrojar luz sobre las circunstancias de representación y composición de una obra que se escribe al calor de las vivencias de Castillo en la ciudad del Turia. Por el camino recalaremos en los vínculos —amistosos y profesionales— entre el autor y algunas de las personalidades del ambiente cultural valenciano; una red de conexiones sobre la que planea la inquietud, plenamente barroca, por la consecución del mecenazgo y la fama.^7^ En el último epígrafe, a fin de aportar una visión unitaria, pondremos en común las hipótesis al respecto de *La fantasma de Valencia* con las fechas que barajamos para *Los encantos de Bretaña* y *El marqués de Cigarral*. De este modo, al circunscribir las obras dentro de un lapso biográfico caracterizado por una especial fruición editorial, podremos establecer algunas consideraciones acerca de los periodos de producción y publicación de Castillo, así como de los derroteros que siguieron sus escritos en el paso a las prensas o, en este caso, a la escena.

## Representola el Valenciano

El índice de *Fiestas del jardín* constituye el primer paso para indagar en la representación de las obras. Allí Castillo asigna a cada una de las comedias su autor correspondiente: *Los encantos de Bretaña*, a Morales; *El marqués del Cigarral*, a Avendaño; y *La fantasma de Valencia*, al Valenciano. Zarpemos pues en busca de este Valenciano y comparemos sus huellas con las del dramaturgo. Castillo recorrería los principales núcleos literarios de la España del xvii al servicio de grandes señores de las familias Benavente-Pimentel y Fajardo-Requesens.^8^ Es así como en 1628, cuando su señor Luis Fajardo, marqués de los Vélez, es nombrado virrey de Valencia, abandona finalmente la Corte para establecerse en la ciudad levantina.^9^ Apenas un año después, en 1629, ven la luz dos de sus nuevas obras: *Lisardo enamorado* y *Huerta de Valencia.* En total, el tiempo que Castillo permanece en las orillas del Turia se concreta en dos periodos: el primero, entre 1628 y 1630; y, el segundo, entre 1634 y 1635. De acuerdo con estas fechas, la estancia en Valencia se interrumpe durante unos años, desde 1631 a 1633, que pasó en Barcelona.^10^ Al abrigo del ambiente de las Academias, el novelista y dramaturgo lograría abonar, como antes en la etapa madrileña, una fértil producción literaria.

En cuanto al autor de comedias, los datos que alberga el DICAT,^11^ herramienta imprescindible en este proceso, revelan en una primera búsqueda tres entradas que confluyen en la etiqueta de este Valenciano*.* Las dos primeras no solo comparten identidad dramática sino semblanza, pues bajo ambos registros se ocultan los gemelos Almella (Almela o Amella), de nombre Juan Bautista y Juan Jerónimo. El origen del apelativo, tal y como deduce Ferrer Valls (2002, 137), vendría determinado por su lugar de nacimiento, la localidad de Morella.^12^ En calidad de actores, hay constancia de que en más de una ocasión pusieron su físico al servicio de la comedia; por ejemplo, en el auto *Los hermanos parecidos* de Tirso, escrita expresamente para ellos y que se representó en Toledo durante la Octava del Corpus de 1615.^13^ Los dos, junto con la mujer del primero, Manuela Enríquez, trabajan como intérpretes en la compañía de Cristóbal Ortiz hasta que, en 1620, el mismo Juan Bautista Almella pasa a asumir la dirección^14^; un cargo que ocupará Juan Jerónimo tras la muerte de su hermano en 1624.

Este primer rastreo permite descartar la posibilidad de que Juan Bautista Almella sea el Valenciano al que Castillo hace referencia en *Fiestas del jardín.* No obstante, si recuperamos la tercera de las entradas que aparecían en los resultados de búsqueda del DICAT, entra en consideración la existencia de un tercer Valenciano, distinto del anterior: Juan Bautista Valenciano. Es probable, así lo afirma Ferrer Valls (2002, 135), que tras el fallecimiento de Almella este actor se apropiase del apodo como una hábil estrategia para valerse de la fama de la que gozaban los gemelos. A partir de aquí, el siguiente paso es dar con aquellas fechas en que uno u otro —recordemos, Juan Jerónimo Almella o, el tercero en discordia, Juan Bautista Valenciano— pudiesen haber pisado las tablas de Valencia*.* A lo largo de los años posteriores a 1624, Juan Jerónimo participa en las celebraciones del Corpus en Córdoba y Sevilla.^15^ En 1627, hay noticia de que la compañía de Juan Bautista, que había representado en la casa de comedias de la Olivera de Valencia el día 22 de julio, no pudo hacerlo el 23 y 24 por falta de público. Ya fuera el apócrifo Juan Bautista o, menos probable, la compañía de Juan Jerónimo aún conocida por el nombre de su hermano, lo cierto es que Castillo Solórzano no abandona la Corte hasta 1628, por lo que no fue *La fantasma de Valencia* la responsable del fracaso de la compañía.

En el caso de Juan Bautista Valenciano, desde 1627 hasta la última fecha que registra el DICAT, 1657, no aparece en ninguna otra ocasión vinculado a la ciudad de Valencia. En 1634, año de edición de *La fantasma,* llevó a cabo once funciones, en periodo indeterminado, en el patio de comedias de Ávila. Aunque la falta de pistas suficientes impide negar con rotundidad que coincidió con Castillo en Valencia, los datos que existen en torno a los movimientos de Juan Jerónimo, y amparado por su popularidad en la época, hacen más verosímil la identificación de este último con el Valenciano de Castillo. Volvamos entonces sobre sus pasos: el 3 de abril de 1628, año en que Castillo deja Madrid, Juan Jerónimo pacta con el apoderado del Hospital General de Valencia, Jacinto Alonso Maluenda, un acuerdo para trasladarse allí desde Ciudad Rodrigo. El propósito era representar sesenta comedias en el edificio teatral de la ciudad, la *casa de comèdies* de la Olivera. Después de un intervalo en el que pasa de Madrid a Segovia, de nuevo lo reciben las tablas de la Olivera en el mes de junio. Seguiría allí los meses de julio y agosto, hasta que, finalmente, en septiembre toma el relevo la compañía de Andrés de la Vega.^16^

Las noticias de 1629 son confusas, pero en 1630 Juan Jerónimo se halla en Murcia como miembro de la compañía del autor Francisco Mudarra. Ya sea como parte de esta o como autor en la suya propia, los nombres de ambos aparecen ligados en los años siguientes. En 1631, momento en que Castillo abandona la ciudad del Turia para trasladarse a Barcelona, un documento del libro de cuentas del Hospital General de Valencia con fecha del 17 de enero atestigua el compromiso de Mudarra y Juan Jerónimo para asumir cuarenta representaciones, que darían inicio el día 25 y se prolongarían hasta el mes de marzo. En 1632, Juan Jerónimo estaba en Córdoba y no hay constancia —al menos, según los datos con que contamos— de que volviese a Valencia en los años anteriores a su muerte, que con seguridad ya se había producido en 1653.

Así pues, si recapitulamos las pinceladas anteriores, estamos en condiciones de aislar dos franjas temporales en las que Juan Jerónimo representa en la ciudad de Valencia: de abril a septiembre de 1628, y del 25 de enero a marzo de 1631. Observar ambos periodos en paralelo a los derroteros de Castillo acota algo más estas consideraciones. Con respecto al año de 1631, como comentábamos, cuando Luis Fajardo —el por aquel entonces marqués de los Vélez— muere el 24 de diciembre, Castillo se encontraba ya ausente, probablemente en Barcelona. Por otra parte, en 1634, año de edición de *Fiestas del jardín* —cuya aprobación data del mes de mayo—, no hay noticia de la presencia de Juan Jerónimo en Valencia*,* que se movía entre las urbes andaluzas de Sevilla y Écija.

Frente a estas incompatibilidades, lo más probable es que Castillo no escribiese la obra en fecha cercana a su edición (1634), sino que lo hiciese durante su primera estancia en Valencia (1628–1631). Aun así, para intentar concretar el abanico temporal en cuanto a las fechas de, primero, composición y, después, representación, resulta conveniente acudir a otros factores antes de elaborar las hipótesis conclusivas.

## Jacinto Alonso Maluenda (¿1597?-1657): un puente entre Castillo y Juan Jerónimo Almella

En el conjunto de los datos extraídos del DICAT, relativos a los avatares biográficos de Juan Jerónimo Valenciano, sale a relucir el nombre de Jacinto Alonso Maluenda. Amigo de Castillo y conocida figura en la sociedad áurea valenciana, en 1622 había heredado de su padre el puesto de alcaide en el teatro ubicado en Vall-Cubert, la actual calle de les Barques (Barrera y Leirado 1860),^17^ que ocupaba desde 1584. Este teatro no es otro que la *casa de comèdies* de la Olivera, que se había inaugurado en 1619, y en cuyas dependencias Maluenda tendría también su residencia, la llamada ‘casa del Autor’.^18^ Junto con la actividad administrativa, se dedicó al oficio de las letras, fundamentalmente a la lírica jocosa. En estas circunstancias se apoya Juliá Martínez (1951, 16) para poner de relieve ‘la gran influencia que ejerció en el ambiente cultural valenciano y sus constantes viajes, que le pusieron en contacto continuo con los escritores de su época, especialmente en Madrid, así como la amistad que tuvo con Castillo Solórzano’.^19^ Este fragmento permite contemplar la posibilidad de que Maluenda hubiese acaso coincidido con Castillo en la Corte. Tanto si fue así como si las presentaciones se llevaron a cabo por vez primera en Valencia, una vez instalado allí Castillo, lo que es seguro es que en 1629 se había establecido entre los dos un lazo de amistad. Lo acredita la décima con la que Castillo contribuye ese mismo año a *Cozquilla del gusto*, una colección de poemas de Maluenda con licencia del mes de mayo. La elogiosa composición se inicia con los siguientes versos: ‘De crítica detracción / seguro, Jacinto, estáis, / cuando con la pluma dais / deleite y admiración’ (*Cozquilla del gusto,* 9). En términos semejantes volvería a participar en *Tropezón de la risa*, publicada en fecha desconocida.

Parece lógico considerar que, dado su papel en el motor teatral de la ciudad y teniendo en cuenta que la contratación de compañías para la Olivera constaba entre sus tareas habituales, Maluenda pudo funcionar como puente entre Castillo y Juan Jerónimo. En 1628, tal y como veíamos en el apartado anterior y de acuerdo con el DICAT, Maluenda viaja a Madrid para reunirse con el Valenciano y concertar con él sesenta representaciones para el corral de la Olivera, en calidad de apoderado del Hospital General. Tal acuerdo acarrearía una serie de catastróficas desavenencias económicas. El adelanto de la suma necesaria concedido por el Hospital a la compañía para sufragar los gastos del viaje y el arreglo de las comedias termina por generar una deuda. El 15 de mayo del mismo año se interpone un requerimiento judicial para encarcelar al autor y embargar sus bienes; pero, finalmente, se resuelve con la asunción del compromiso por parte de Juan Jerónimo de restituir la cifra.

La anécdota —además del interés que por sí sola pueda despertar— evidencia las conexiones entre la compañía de Juan Jerónimo, el Hospital y Jacinto Maluenda; pero, además, confirma la presencia del autor de comedias en la ciudad en el mes de mayo de 1628. En esta época, Castillo se hallaba con seguridad en Valencia, pues su primera obra allí publicada, *Lisardo enamorado*,^20^ recibió la aprobación en este mismo mes, aunque no se diese a la imprenta hasta el siguiente año. Otro documento corrobora que Juan Jerónimo se encontraba ya en Madrid para el día 26. Desde allí, como sabemos, volvería a Valencia para representar en la Olivera durante los meses de junio, julio y agosto, hasta el 1 de septiembre. Como dato significativo, no puede dejar de comentarse el depósito de todo un conjunto de comedias y vestuario teatral que el 14 de junio Almella entrega al clavario del Hospital a modo de garantía por el préstamo de cierta suma. Tras consultar con atención la lista de títulos, que se conserva en una escritura del 9 de agosto, constatamos que no está entre ellas *La fantasma de Valencia,* como ninguna otra de Castillo Solórzano.^21^

A pesar de este último dato, que retomaremos en el apartado siguiente, prevalece el hecho de que Juan Jerónimo había permanecido en la ciudad —con el paréntesis de un mes— de mayo a septiembre. Si tenemos en cuenta que Castillo se había mantenido activo en los círculos literarios de las academias de Valencia, en las que participaba en compañía de Maluenda, y su vivo interés por la dramaturgia, no sería de extrañar que hubiese estado al tanto de las idas y venidas de su amigo, así como de sus actividades relativas a la gestión de la Olivera. En cualquier caso, la relación entre los dos se mantenía en 1631, cuando en el mes de enero Maluenda viaja a Murcia de nuevo en busca de Mudarra.

Tal y como había hecho Castillo años atrás, Maluenda participa en 1634 en la edición de *Fiestas del jardín* y lo retribuye con la décima siguiente:

Hasta el último confín

lleva de este jardín flores

la fama, con superiores

aplausos de su clarín,

de heroico estilo, en jardín,

tales fiestas (siendo solo

desde el uno al otro Polo

vuestro ingenio en gloria tanta).

Si la envidia las quebranta,

yo sé que las guarda Apolo (*Fiestas del jardín*, 88).

## Dos hipótesis

*La fantasma de Valencia* tuvo ocasión de caer en manos de Juan Jerónimo en cualquiera de las dos etapas (1628 y 1631) en que el autor de comedias coincidió con Castillo. Si se representó en 1628, el dramaturgo tendría que haberla escrito forzosamente durante los primeros meses de su estancia. Por el contrario, si sucedió en 1631, habría dispuesto de unos tres años para familiarizarse primero con el mapa de la urbe, su imaginario y su poso cultural. Si bien es cierto que pudo haberla compuesto en Barcelona, el género de *La fantasma* no encaja con el de las que allí se editaron, pues tanto *Las harpías en Madrid* (1631), *La niña de los embustes* (1632) y *Los amantes andaluces* (1633) contienen, todas ellas, novelas. Sí habría resultado coherente insertarla en *Noches de placer* (1631), cuyas narraciones están dedicadas a distintas personalidades del territorio valenciano; pero el hecho es que no se publica. La sucesión más lógica de acontecimientos conduce a que, de vuelta en Valencia en 1634, Castillo decide recuperar *La fantasma de Valencia* y otras dos comedias aún inéditas —pero ya representadas— e insertarlas una colección que conformaría ‘una relación novelada de una fiesta del siglo XVII’ (Festini, 2011, 213). De haberse vendido la obra a Juan Jerónimo, cabría objetar, no habría sido posible obtener licencia de impresión. Sin embargo, en la aprobación de *Fiestas del jardín* a cargo de fray Felipe Salazar, se lee el siguiente pasaje, muy revelador:

Las comedias son suyas [de Castillo], no solo por haberlas compuesto, *sino por no haberlas vendido*; punto en que se debe reparar, porque imprimir las que han comprado los que representan, sin su gusto, no sé que pueda hacerse sin escrúpulo, pues es manifiesta injusticia y en perjuicio del poseedor legítimo (Castillo Solórzano, *Fiestas del jardín*, 85)^22^

Según esto, la contingencia de que Castillo hubiese cedido —que no vendido— la obra no deja de apoyar la hipótesis de una fecha de composición anterior a 1634. Que no fuera de su propiedad explicaría, además, por qué Juan Jerónimo no pudo dejarla en depósito al clavario del Hospital General en junio de 1628 junto con el resto de las obras. Así, podemos sostener que, en el periodo de 1628 a 1631, Castillo vierte el retrato de la Valencia en la que vive en forma de comedia. Desde aquí, es razonable establecer dos hipótesis:

- El mismo año de su llegada a Valencia, 1628, compone la obra y la entrega a Juan Jerónimo Almella, el Valenciano. Pudo representarse en la Olivera en algún momento entre abril y septiembre.
- A principios de 1631, sabiendo que Juan Jerónimo se encuentra de nuevo en la ciudad, le cede la comedia, que ha escrito en los años anteriores. Se representa en la Olivera en algún momento entre el 25 de enero y el mes de marzo.

Cabe contemplar la opción de que *La fantasma de Valencia* viese la luz *ad hoc* para que la compañía de Juan Jerónimo la llevase a escena. También es probable que ambos entrasen en contacto a través de la mediación de Jacinto Alonso Maluenda. En uno u otro caso, asumimos que la comedia o bien se copió o bien, una vez representada, se devolvió al dramaturgo o se depositó tal vez en la Olivera al cuidado de Maluenda. Es poco probable, por lo tanto, que Juan Jerónimo la guardase para realizar más representaciones. Sea como fuere, en 1634 aún pertenecía a Castillo, según se colige de la declaración de Felipe Salazar. Finalmente, el mismo año de 1634, tras abandonar Barcelona y de regreso en Valencia, Castillo reclama la comedia y la inserta en el volumen de inspiración valenciana *Fiestas del jardín,* que consigue la licencia el 4 de mayo.

### Consideraciones literarias acerca de la fiesta de San Juan

Frente a la perspectiva documental adoptada hasta ahora, una aproximación de corte más literario a la comedia podría servir de refuerzo a las consideraciones anteriores. El argumento de *La fantasma de Valencia* transcurre en el día de San Juan; es decir, el 24 de junio, durante el solsticio de verano.^23^ Si bien es cierto que los ingenios áureos encontraron a menudo un socorrido escenario para sus comedias en esta celebración, que ofrecía unas coordenadas semejantes al enredo carnavelesco,^24^ no podemos dejar de abordar la posibilidad de que Castillo Solórzano escribiera la obra para la ocasión. De existir una correspondencia entre el tiempo ficcional y referencial, podría acotarse un periodo más ajustado alrededor del 24 de junio, fecha en la que la compañía de Juan Jerónimo pisaba, precisamente, el tablado de la Olivera. Bajo el signo de la fiesta urbana, es verosímil que la comedia se concibiese para una representación en el exterior; hipótesis que se apoya en la extensión del texto, que cuenta con alrededor de 830 versos por acto, cuantitativamente menor a la habitual en el teatro del xvii.^25^ Quizá esto sirva para justificar el hecho de que *La fantasma de Valencia* no se vendiese al autor, puesto que habría nacido específicamente para una ocasión efímera.

Si, en efecto, la comedia se llevó a las calles valencianas, el fragmento narrativo que enmarca el conjunto de la tercera fiesta puede tal vez interpretarse como una descripción inspirada en estas circunstancias de representación: ‘habiendo los caballeros y damas acabado de comer […] se pusieron a las ventanas que caían a la calle y en los cadalsos que abajo estaban, donde hallaron más de veinte lucidos caballeros puestos en hermosos y bien enjaezados caballos’ (Castillo Solórzano, *Fiestas del jardín*, 217). Y más adelante: ‘Acabada [la colación], todos tomaron asientos en torno del teatro, el cual estaba lucidamente adornado todo de flores artificiales y volantes de plata’ (*Fiestas del jardín*, 217). El pasaje se hace eco de los divertimentos propios de San Juan y otras fechas señaladas, ocasiones en las que las calles se verían engalanadas con improvisados escenarios. En uno de estos tablados, o *cadalsos*, bien pudo haber tenido lugar la representación de *La fantasma de Valencia*.^26^

Otros motivos sanjuanescos aderezan la ambientación de la obra. Apenas iniciada, dos damas se asoman al balcón siguiendo una coqueta costumbre de San Juan que consistía en indagar el nombre de posibles pretendientes: ‘Si acabaste la oración, / Fenisa, para saber / del novio que has de tener / el nombre, ponte al balcón / y al que pasare primero / pregunta cómo se llama’ (Castillo Solórzano, *La fantasma de Valencia*, vv. 21–26).^27^

## Sobre la fecha de composición de *Los encantos de Bretaña y El marqués del Cigarral*

Pongamos de nuevo nuestra atención en *Fiestas del jardín* y volvamos sobre las otras dos comedias que acompañan a *La fantasma de Valencia*, al objeto de delimitar el lapso temporal en que se escribieron. De *Los encantos de Bretaña* nos dice Castillo que la llevó a escena Morales, quien, asumimos, se trata del autor de comedias Juan de Morales Medrano. Sabemos que su compañía estuvo trabajando en Valencia, al menos, desde diciembre de 1629 hasta julio de 1630 y, de nuevo, entre abril y julio de 1631, como reemplazo a la compañía del Valenciano. En enero de 1632, Morales ‘disolvió su compañía para entrar con su mujer, Josefa Vaca, en la dirigida por Antonio de Prado en virtud del casamiento convenido entre éste y Mariana Vaca de Medrano, hija de Juan Morales’ (DICAT). Por lo tanto, la posibilidad de coincidencia con Castillo termina en julio de 1631. Conviene señalar, asimismo, que *Los encantos de Bretaña* es una adaptación de *La cautela sin efecto,* novela del propio Castillo Solórzano que se recoge en *Noches de placer,* libro publicado en Barcelona en 1631, con aprobaciones de febrero de tal año. Si la comedia se compuso con posterioridad a la publicación de *Noches de placer,* el margen temporal se estrecha lo suficiente como para quedar delimitado entre abril y julio de 1631, coincidiendo con la última estancia en Valencia de la compañía de Morales.

La tercera de las comedias que aparece en *Fiestas del jardín* es *El marqués del Cigarral,* probablemente la más célebre dentro del corpus dramático de Castillo Solórzano junto con *El mayorazgo figura*,^28^ y representada, de acuerdo con el índice, por Avendaño. Los datos recogidos en el DICAT sitúan a la compañía de Avendaño en Valencia entre julio y octubre de 1631, de modo que habría sustituido a la compañía de Morales en las tablas de la Olivera durante la segunda mitad de aquella temporada. Así, los tres autores cuyas compañías Castillo asegura que llevaron a escena las comedias contenidas en *Fiestas del jardín* trabajaron en Valencia entre enero y octubre de 1631 de forma consecutiva: primero la del Valenciano —de enero a marzo—, después la de Morales —de abril a julio—, y, por último, la de Avendaño —de julio a octubre—.

La fecha de publicación de *Noches de placer* podría aproximar la fecha de composición y representación hasta ese año de 1631 en que coincidieron en Valencia las tres compañías. Queda la duda de cuáles pudieron ser las circunstancias en las que se llevaron a escena, ya que no parece que las comedias entraran en el repertorio de ninguna de las compañías. ¿Quizás se representaron en un contexto de celebración cortesana, del estilo de las utilizadas por el propio Castillo Solórzano como marco para sus colecciones? Sea como fuere, las tres encontraron acomodo precisamente en la siguiente colección de Castillo, ya que no en los libros más propiamente novelescos, en los que sí que supo encajar, en cambio, sus obras de teatro breve: *El comisario de figuras* aparece en *Las harpías en Madrid* (1631); *El barbador* y *La prueba de los doctores*, en *La niña de los embustes* (1632); y *La castañera*, en *Aventuras del bachiller Trapaza* (1637).^29^

Pero el DICAT nos ofrece aún otros datos sobre las actividades de Avendaño en Valencia que no podemos obviar. La *troupe* de Avendaño volvió a ser contratada para representar en la Olivera en 1632, desde el 11 de abril hasta el 31 de mayo, aunque su estancia se prolongaría hasta mediados de julio. Estaban allí, por lo tanto, durante la visita de Felipe IV a la ciudad, que comenzó el lunes 19 de abril y cuyas celebraciones se prolongaron hasta el domingo siguiente. Durante esa semana se celebraron en la ciudad no menos de cinco comedias, y una de ellas, la del martes 20 de abril, se representó en el palacio virreinal. Precisamente, el virrey en aquel momento era Pedro Fajardo, quinto Marqués de los Vélez, quien había heredado el puesto de su padre, Luis Fajardo, unos meses antes, y a cuyo servicio como maestresala continuaría nuestro Alonso de Castillo Solórzano. ¿Sería *El marqués del Cigarral* la comedia representada ante Felipe IV en el palacio virreinal?

Más allá de cualquier hipótesis, resulta evidente que Castillo Solórzano encontró en Valencia un terreno fecundo para su teatro. Alrededor de la mitad del corpus dramático total del autor se concentra entre 1629 y 1635 y en torno a la ciudad levantina. Más todavía si a las obras que hemos examinado en este trabajo añadimos un nuevo título: *La victoria de Norlingen*.^30^ En cuanto a las obras de *Fiestas del jardín,* el rastro de su puesta en escena y de aquellos aplausos que habían despertado entre los espectadores nos ha permitido ahondar en los ciclos de escritura del dramaturgo, establecer periodos de producción significativos y, al cabo, mientras seguimos el peregrinaje de las compañías teatrales a lo largo de la Península, acotar la datación de estas comedias, las únicas representadas —que sepamos— de Castillo Solórzano.

## Footnotes

1 Citamos siguiendo la reciente edición de *Fiestas del jardín*, a cargo de Fuentes Nieto (2019).

2 En palabras de Festini: ‘La estructura guarda una marcada relación con los *Cigarrales* tirsianos, puesto que el marco es una estructura sintagmática que genera la inclusión de nuevas unidades’ (2011, 213).

3 Lo que se incluye en *Tiempo de regocijo* es el entremés de *El casamentero*. Se trata de la primera ocasión en que el autor inserta una pieza dramática. Existe una edición de Cotarelo (1907) publicada junto a *Las harpías en Madrid* en el tomo VII de su *Colección selecta de antiguas novelas españolas*.

4 A la inversa, en un práctico diálogo entre géneros, era habitual que la materia teatral nutriese la novela corta. En su estudio introductorio al respecto de otra de las colecciones del autor, *Noches de placer*, señala Giorgi (2013, 33): ‘las historias se articulan a menudo con un esquema teatral (la división en tres jornadas de la comedia nueva) y los protagonistas también parecen corresponder a los tipos fijos de la comedia, puestos en escena y liquidados en virtud de su funcionalidad; por último, no faltan algunas indicaciones sobre sus gestos antes de hablar o, incluso, glosas sobre la mímica facial’. El fenómeno de las reescrituras está a la orden del día en los siglos áureos y Castillo Solórzano lo practicó a menudo. Véase Giorgi (2014) y Fernández Rodríguez (2020).

5 La última edición crítica de *Los alivios de Casandra*, publicada en 2020,corre a cargo de Andrea Bresadola.

6 *La fantasma de Valencia* comparte título —no así argumento— con una novela que Castillo Solórzano incluyó en la colección *Tardes entretenidas* (1625).

7 Tanto el corpus como la biografía, o aun el carácter mismo de Castillo, se han analizado a la luz de esta búsqueda del doblete áureo que aspiraba a aunar sustento con reconocimiento. Al servicio de figuras notables, el desfile por los principales núcleos culturales de la geografía española se materializa en forma de abundantes dedicatorias a personalidades políticas (Cayuela y Gandoulphe 1999) u obras —como la que nos ocupa— de inspiración local; al tiempo que le permite colarse en composiciones de amigos y valedores como Lope, que lo menciona con elogios en su *Laurel de Apolo*. No es poco lo que se ha dicho sobre esta cuestión. Remitimos a Monzó (2017a), a las recientes publicaciones de González Ramírez (2019) y de Collantes Sánchez, Özmen y Ruiz Pérez (2019); y, como estudio de referencia, a Vélez (2006).

8 En su introducción a *Los amantes andaluces*, Margherita Mulas (2020, 13–23) expone detalladamente los lazos familiares existentes entre los nobles a los que sirvió Castillo Solórzano, y aborda cómo el servicio a sus respectivas casas influyó en la vida y obra del escritor.

9 Para la etapa de Castillo en Valencia y su relación con el marquesado de los Vélez, véase también Monzó (2017b).

10 Cotarelo (1906) sostiene la posibilidad de que Castillo dejase de servir a los Fajardo durante los años de Barcelona, pues el dramaturgo se hallaba ausente en el momento en que murió Luis Fajardo en Valencia en 1631. Asimismo, apunta a las ventajas que la capital de Cataluña podría haberle ofrecido a la hora de publicar sus nuevas comedias (1906, 47). Mulas (2020, 17) propone como residencia del escritor entre 1631 y 1633 el Palau Menor de Barcelona, propiedad de Luis Fajardo como marqués de Martorell. No obstante, lo cierto es que extraña que durante un periodo de tiempo tan largo como el que supuestamente vivió en Barcelona, Castillo Solórzano no desarrollase una vida social y cultural tan intensa como en Madrid, Valencia o Zaragoza; o que, si lo hizo, no haya dejado huellas en sus libros. Si algo podemos deducir de las dedicatorias de sus libros publicados durante esta etapa levantina es que todas van dirigidas a personajes de la aristocracia valenciana, incluyendo los que aparecieron en Barcelona. Así, aunque *Noches de Placer* se abre con un *encomium urbis* de Barcelona, los nobles dedicatarios de las novelas cortas que contiene forman parte de la corte valenciana (Cayuela y Gandoulphe 1999; Giorgi 2013): *Las harpías en Madrid* va dedicada a Francisco Maza de Rocamora; *La niña de los embustes*, a Juan Alfonso Martínez de Vera, teniente de baile de la ciudad de Alicante; y *Los amantes andaluces* va sin dedicatoria. De 1633 data la noticia encontrada en el DICAT de un documento en el que figura Castillo Solórzano como procurador de Pedro de Ortegón, autor de comedias de Barcelona, en cuyo nombre negocia con el clavario del hospital de Valencia la contratación de la compañía. Si bien no son datos concluyentes, parecen apuntar más a una relación comercial o editorial con Barcelona que a una estancia continuada en la ciudad.

11 Ferrer Valls (dir.) (2008).

12 Ferrer Valls (2002) resuelve una confusión por la que se consideraba la existencia de dos autores distintos: Juan Jerónimo Almella, por un lado, y Juan Jerónimo Valenciano, por otro. Ferrer determina que se trata, en realidad, de la misma persona y así, efectivamente, aparece en el DICAT. Remito al artículo para más detalles sobre esta interesante cuestión.

13 Una de las novelas de Castillo incluidas en *Fiestas del jardín* coincide en título con el auto de Tirso; sin embargo, es lo único que tienen en común.

14 Como curiosidad, parece ser que fue una representación de los Valenciano de una obra de Andrés de Claramonte la que provocó el incendio del Coliseo de Sevilla en julio de 1620.

15 Para más detalles acerca de la actividad teatral de Juan Jerónimo en Sevilla, véase Bolaños (2006).

16 A este periodo de representación hay que añadir la excepción de los días 1, 3, 4, 15 y 29 de julio, y 12, 14 y 21 de agosto, en que no hubo representación.

17 Quizá este teatro se refiera a la *Casa dels Santets*, el segundo espacio teatral de la ciudad junto con la casa de comedias de la Olivera, aunque se hallaba situada cerca de la actual plaza de Sant Vicent Ferrer. Sirera (1986, 28) hace referencia al Hostal del Gamell, del que nos dice: ‘desde 1577 a 1598 por lo menos albergó en su patio representaciones de farsas y saltimbanquis. Y es muy posible que este hostal no fuese ni el primero ni el único: sabido es que las primeras compañías profesionales […] actuaban en estos lugares públicos y siempre concurridos’.

18 Arellano (1987, 9) apunta a una continuidad del cargo por parte de los descendientes de Maluenda: ‘La familia parece seguir desempeñando estas funciones en la Casa de la Olivera: en 1673 es alcaide su hijo Luciano Maluenda, y en 1684 se nombra en un documento a la “alcaidesa” Margarita Maluenda’. En cuanto a la historia de la Olivera, principal espacio teatral de la ciudad que se había erigido sobre la planta del antiguo edificio, consúltese Sirera (1986) y Mouyen (1991). Recientemente, el profesor Joan Oleza ha dirigido un proyecto de reconstrucción de la casa de comedias.

19 Juliá Martínez (1951, 16) lo ubica, además, ‘entre los lopistas’ y comenta cómo Maluenda protestó al encontrarse excluido del *Laurel de Apolo* de Lope.

20 Fue el primer libro que publicó en Valencia, pero no sería exacto decir que fue el primero que escribió, ya que se trata de una reescritura de *Escarmientos de amor moralizados,* probablemente más cercana a lo que habría querido publicar el autor originariamente. Es posible que en la elaboración de esta obra influyera la prohibición del Consejo de Castilla de otorgar licencias de impresión para teatro y novelas entre 1625 y 1634. Quizás Castillo se viera forzado a crear una estructura que, en cierto modo, desvirtúa el desarrollo narrativo del libro y la experiencia y disfrute de la lectura, para lograr soslayar la prohibición: el libro se divide en breves capítulos que narran un asunto amoroso, cada uno de ellos seguido de un riguroso examen de todos los errores morales —es decir, el *escarmiento*— que los personajes han cometido. En este sentido, llama la atención lo meticuloso y prolijo de la licencia real, que aparece en *Escarmientos*, y que ocupa nada menos que tres páginas. Parece que don Sebastián de Contreras, quien la firma en nombre del rey, puso especial cuidado en explicar por qué se daba licencia de impresión a un libro al que, dada su naturaleza novelesca y por mucho que Castillo intentara camuflarla, debía haberse negado. En cualquier caso, es de suponer que apenas puso un pie fuera de Castilla, nuestro autor se aplicase a la tarea de rescatar y reescribir su *Lisardo enamorado,* ya puramente novelesco y sin rastro de aquellas extrañas moralinas. Esta misma cuestión de la prohibición avala la posibilidad de que *El agravio satisfecho*, adaptación teatral de la novela ejemplar cervantina *La fuerza de la sangre* que se incluye en *Huerta de Valencia*, pudiera haber sido escrita también anteriormente. Para la conexión entre la novela de Cervantes, la comedia homónima de Guillén de Castro, y la reescritura de Castillo Solórzano, véase Escudero Baztán (2013). Asimismo, para más información sobre la prohibición de licencias para novelas, véase Cayuela (1993, 59–60), que ofrece algunos ejemplos de las huellas que dejó en los preliminares de los libros del autor.

21 Ferrer Valls (2002, 20–21) reproduce los títulos de esta lista.

22 La cursiva es nuestra.

23 La fiesta de San Juan impregna el contexto festivo en que se desarrollan los compases iniciales de la obra y permite introducir una serie de motivos de raigambre popular. El primer verso de la comedia, ‘Alegre noche’, funciona como preludio y adelanta el tono desenfadado que marca el desarrollo del primer acto.

24 Según Caro Baroja (1979, 123), la festividad ‘ha dado motivo a miles de versos, refranes y comentarios. Es la fiesta más popular de todas. Su universalidad era conocida de los poetas de otros tiempos’. En esta noche se inspira Lope, por ejemplo, en *El poder en el discreto*, *La noche de San Juan* o *La malcasada*.

25 Sobre la extensión promedio afirma Mata Induráin (2001): ‘frente a los 3.000 versos de que consta aproximadamente una comedia típica, la burlesca tiene un promedio de 1.800, un poco más de la mitad’.

26 Sirera (1986, 26) señala: ‘Menos documentados, al menos hasta entrado el siglo XVI, están los escenarios fijos, adosados a algún edificio; a partir de ese momento el procedimiento se generalizará no sólo para representar obras religiosas sino también otras de tipo conmemorativo. En el campo del teatro profano, calles, plazas y descampados son los lugares idóneos para representaciones de tipo para-teatral o folklórico’.

27 Deleito y Piñuela (1944, 54) documenta este tipo de tradiciones populares: ‘Con frecuencia, las doncellas se ponían a medianoche en las rejas o en los balcones de sus casas, con el cabello suelto y el pie izquierdo dentro de una bacía llena de agua, para averiguar si habían de casarse o no. Si alguno que por allí pasaba decía un nombre, dábanle una cinta para poderle reconocer a la siguiente mañana’.

28 En cuanto al papel que desempeña el dramaturgo en el desarrollo de la comedia de figurón, remitimos al estudio introductorio de Arellano (1989, 33–36) para *El mayorazgo figura*.

29 Para un estudio sobre el uso que hace Castillo Solórzano de los entremeses como piezas intercaladas en sus obras narrativas extensas, véase Fernández Nieto (1983). No deja de ser curioso que, si se mantienen las fechas hipotéticas de redacción de las comedias de *Fiestas del jardín,* nos encontramos con tres comedias y tres entremeses en el mismo periodo. Quizás, alrededor del año 1631, diseñó tres fiestas teatrales y reaprovechó los materiales textuales en los libros publicados durante esa etapa, de un modo similar a como lo haría habitualmente; es decir, injiriendo las comedias en obras misceláneas. Tal es el caso de *El agravio satisfecho*, inserto en *Huerta de Valencia* (1629); *El mayorazgo figura*, en *Los alivios de Casandra* de 1640; o *La torre de Florisbella*, en *Sala de recreación*, publicado en 1649; y los entremeses engarzados entre los capítulos de sus novelas.

30 Compartimos con Mª Soledad Arredondo (2006) la hipótesis de que *La victoria de Norlingen* pueda pertenecer también a este ciclo valenciano, así como haberse representado en Valencia. Esto explicaría la relevancia que se da en la obra a Baltasar de Marradas (a quien también se alude brevemente en *La fantasma de Valencia*), figura importante en la Guerra de los Treinta Años, pero que no participó en la batalla de Nördlingen. Algunas de sus hazañas y méritos militares se enumeran muy elogiosamente en una relación en romance mediado el primer acto. Se conocen dos testimonios de *La victoria de Norlingen*: una suelta sin lugar ni año y el recogido en la *Parte 28 de nuevas escogidas* (1667). En ambos se la titula ‘comedia famosa’. La batalla de Nördlingen tuvo lugar a finales del verano de 1634 y poco después circulaban ya las primeras relaciones impresas. Consideramos que esta obra —como *El primer blasón del Austria* de Calderón y *Los dos Fernandos de Austria* de Coello— debió de ser compuesta entre 1634 y 1635. Es razonable, por lo tanto, que la obra se llevase a escena en un contexto celebrativo y propagandístico, ya en el teatro, ya en algún salón o jardín palaciego. El mismo Marqués de los Vélez, que sería nombrado posteriormente mayordomo mayor del Cardenal-Infante don Fernando de Austria, figura protagonista en la batalla y en la obra, podría haber encargado la composición y representación de la comedia.
